# An Emerging Strategy for Neuroinflammation Treatment: Combined Cannabidiol and Angiotensin Receptor Blockers Treatments Effectively Inhibit Glial Nitric Oxide Release

**DOI:** 10.3390/ijms242216254

**Published:** 2023-11-13

**Authors:** Sigal Fleisher-Berkovich, Veronica Battaglia, Francesca Baratta, Paola Brusa, Yvonne Ventura, Nitzan Sharon, Arik Dahan, Massimo Collino, Shimon Ben-Shabat

**Affiliations:** 1 Department of Clinical Biochemistry and Pharmacology, Ben-Gurion University of the Negev, P.O. Box 653, Beer-Sheva 8410501, Israel; yventura@bgu.ac.il (Y.V.); nitzan.sharo@gmail.com (N.S.); arikd@bgu.ac.il (A.D.); 2 Department of Drug Science and Technology, University of Torino, 10124 Torino, Italy; battaglia.veronica91@gmail.com (V.B.); francesca.baratta@unito.it (F.B.); paola.brusa@unito.it (P.B.); 3 Department of Neuroscience “Rita Levi Montalcini”, University of Torino, 10124 Torino, Italy; massimo.collino@unito.it

**Keywords:** microglia, nitric oxide, lipopolysaccharide, cannabidiol, telmisartan

## Abstract

Cannabidiol (CBD), the major non-psychoactive phytocannabinoid found in cannabis, has anti-neuroinflammatory properties. Despite the increasing use of CBD, little is known about its effect in combination with other substances. Combination therapy has been gaining attention recently, aiming to produce more efficient effects. Angiotensin II activates the angiotensin 1 receptor and regulates neuroinflammation and cognition. Angiotensin receptor 1 blockers (ARBs) were shown to be neuroprotective and prevent cognitive decline. The present study aimed to elucidate the combined role of CBD and ARBs in the modulation of lipopolysaccharide (LPS)-induced glial inflammation. While LPS significantly enhanced nitric oxide synthesis vs. the control, telmisartan and CBD, when administered alone, attenuated this effect by 60% and 36%, respectively. Exposure of LPS-stimulated cells to both compounds resulted in the 95% inhibition of glial nitric oxide release (additive effect). A synergistic inhibitory effect on nitric oxide release was observed when cells were co-treated with losartan (5 μM) and CBD (5 μM) (by 80%) compared to exposure to each compound alone (by 22% and 26%, respectively). Telmisartan and CBD given alone increased TNFα levels by 60% and 40%, respectively. CBD and telmisartan, when given together, attenuated the LPS-induced increase in TNFα levels without statistical significance. LPS-induced IL-17 release was attenuated by CBD with or without telmisartan (by 75%) or telmisartan alone (by 60%). LPS-induced Interferon-γ release was attenuated by 80% when telmisartan was administered in the absence or presence of CBD. Anti-inflammatory effects were recorded when CBD was combined with the known anti-inflammatory agent dimethyl fumarate (DMF)/monomethyl fumarate (MMF). A synergistic inhibitory effect of CBD and MMF on glial release of nitric oxide (by 77%) was observed compared to cells exposed to MMF (by 35%) or CBD (by 12%) alone. Overall, this study highlights the potential of new combinations of CBD (5 μM) with losartan (5 μM) or MMF (1 μM) to synergistically attenuate glial NO synthesis. Additive effects on NO production were observed when telmisartan (5 μM) and CBD (5 μM) were administered together to glial cells.

## 1. Introduction

Neuroinflammatory pathways stimulate central resident immune cells (e.g., microglia) and enhance the infiltration of cells from the periphery into the brain [1]. Microglia are identified as the brain’s macrophages and, therefore, can rapidly undergo activation in response to pathological changes in the brain [2,3]. Microglia dually mediate neuroinflammation. On the one hand, microglia migrate toward the injury site after brain injury and exacerbate tissue injury by producing cytotoxic substances such as nitric oxide (NO) and inflammatory cytokines [4]. Evidence supports the neurotoxic role of glia-secreted NO in neuronal death in vitro [5]. Glial inducible nitric oxide synthase (iNOS) has been linked to neurodegeneration [6,7]. On the other hand, microglia also have a role in tissue repair by clearing debris and secreting anti-inflammatory cytokines and growth factors [8,9]. The dual role of microglia is associated with different states of microglial polarization in different cellular contexts [10,11].

Cannabidiol (CBD) is a nonpsychoactive phytocannabinoid that has anti-neuroinflammatory [12,13] and neuroprotective effects [14]. More specifically, several groups have shown that microglial cell functions are affected by CBD. For example, Kozela et al. 2010 showed that CBD inhibits the lipopolysaccharide (LPS)-induced nuclear factor kappa B (NF-κB) interferon β/signal transducers and activators of transcription (IFNβ/STAT) and neuroinflammatory pathways in a cannabinoid receptor type 1 (CB1R)/CB2R-independent manner [15]. As also shown by Torika et al. 2016, neuroinflammation is regulated by angiotensin 1 receptor (AT1R) [16]. Angiotensin II (Ang II) acts primarily via activation of AT1R and regulates blood pressure [17,18]. This receptor has been identified in rat and monkey glial cells (microglia and astrocytes) [19].

Overactivity of AT1R in neurodegenerative diseases enhances glial injury and oxidative damage. Importantly, in several studies, AT1R blockers (ARBs) were shown to be neuroprotective and prevent cognitive decline [20]. Furthermore, the effects of ARBs like sartans (losartan, candesartan and telmisartan) have been presented in primary neuronal and glia and various rodent models of brain diseases related to neuroinflammation [21,22,23]. Additionally, Ang II is also known to activate numerous nuclear transcription factors, such as NF-κB, and inflammatory markers, like the NOD-like receptor protein 3 (NLRP3) inflammasome [24,25,26,27], which is a multi-protein complex composed of NLRP3, the adaptor apoptosis-associated speck-like protein containing a caspase recruitment domain (ASC) and pro-caspase 1. Among the inflammatory mechanisms triggered by LPS, the activation of the NLRP3 inflammasome has attracted considerable attention [27].

It has been shown, for example, that LPS triggered NF-κB activation, a mechanism responsible for iNOS expression in C6 glial cells [28]. Telmisartan reversed the promoting effects of LPS on the protein levels of TLR4, MyD88, and p-p65/p65 (NF-κB), and the inhibitory effect on the PPARγ level in lung tissues from LPS-treated rats [29]. CBD was linked to inflammatory signaling pathways, such as the MAPK pathway (Dusp1, Dusp8, Dusp2), cell cycle-related signaling (Cdkn2b, Gadd45a), as well as JAK/STAT regulatory molecules (Socs3, Cish, Stat1) in LPS-treated BV2 cells [30]. This concept combines two therapeutic agents acting on different upstream pathways but affecting similar downstream factors.

Co-administration of CBD and another cannabinoid, tetrahydrocannabinol (THC), has already produced a better therapeutic profile than each phytocannabinoid alone [31,32,33]. This synergism between the inflammation related to the two cannabinoids has been reported in vivo. A more efficient decrease in microglial activation in Alzheimer’s transgenic mice was observed upon THC and CBD treatment than after treatment with either THC or CBD alone [34]. A similar combination of phytocannabinoids was shown to be neuroprotective in a Huntington’s disease model involving inflammation. Both CB1 and CB2 receptors were shown to be involved in the anti-inflammatory actions of the combined cannabinoid therapy [35].

The present study aimed to elucidate the combined role of AT1R blockers (ARBs) and CBD in the modulation of glial inflammation. To achieve this goal, the specific aims of the present study were: A. to investigate the combined effect of CBD and ARBs (e.g., telmisartan, losartan and candesartan) on NO release from BV2 murine microglial cells; B. to investigate the combined effect of CBD and telmisartan on cytokine released from BV2 cells; C. to compare the efficacy of ARBs as inhibitors of glial inflammation to DMF, a drug with immunomodulatory properties; and D. to follow the regulation of inducible NLRP3 inflammasome expression by telmisartan and CBD in BV2 cells.

## 2. Results

The combined effects of CBD with drugs that had previously been shown to act as anti-neuroinflammatory agents were checked here for additive or synergistic effects.

**Figure 1:**
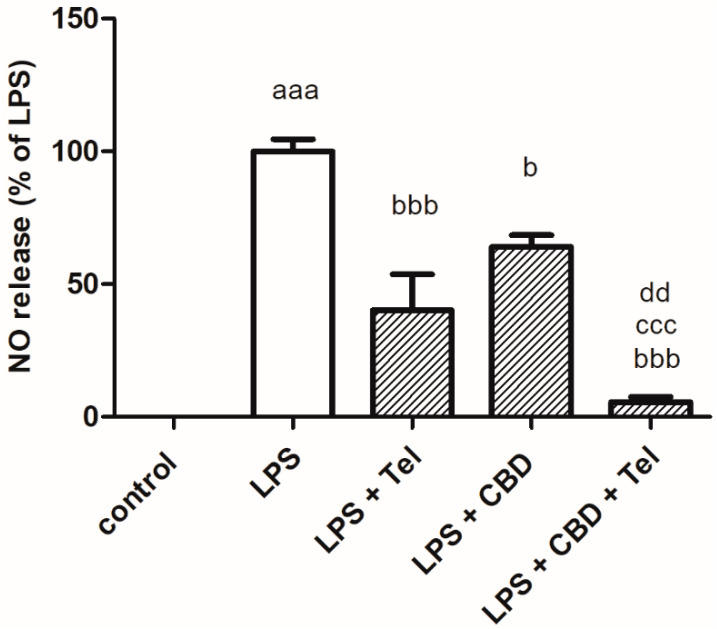
Telmisartan and CBD additively decreased NO production in LPS-stimulated BV2 microglia. BV2 microglia were incubated with LPS (7 ng/mL), LPS + telmisartan 5 μM, LPS + CBD 5 μM or LPS + CBD 5 μM + telmisartan 5 μM for 24 h. NO levels were determined in the media and normalized to the cell number. Three independent experiments were performed, *n* = 12; ^aaa^ *p* < 0.001 vs. control (non-stimulated cells); ^bbb^ *p* < 0.001 vs. LPS; ^b^ *p* < 0.05 vs. LPS; ^ccc^ *p* < 0.001 vs. LPS + telmisartan 5 μM; ^dd^ *p* < 0.01 vs. LPS + CBD 5 μM.

We examined the synthesis of NO as a marker for neuroinflammation in BV2 microglia stimulated with the inflammation inducer LPS (7 ng/mL) [16,36,37,38] and treated with ARB, telmisartan (5 μM), or CBD (5 μM) alone or in combination. While LPS significantly enhanced NO synthesis compared to non-stimulated cells (control), telmisartan and CBD attenuated this effect by 60% and 37%, respectively. Exposure of LPS-stimulated cells to both compounds resulted in an additive inhibitory effect (decreased NO synthesis by 95%) (Figure 1).

**Figure 2:**
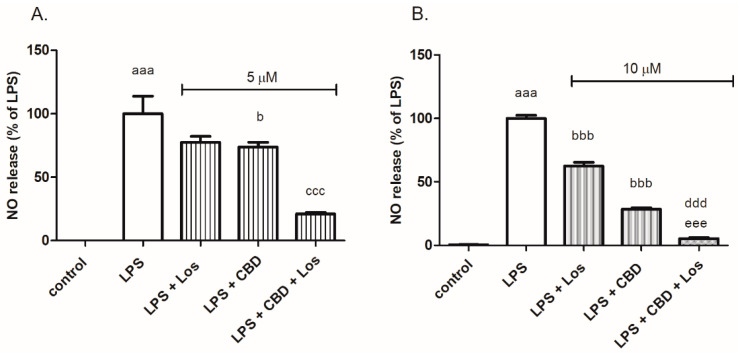
Losartan and CBD synergistically or additively decreased NO production in LPS-stimulated BV2 microglia, depending on the CBD concentration used. BV2 microglial cells were incubated with LPS 7 ng/mL, LPS 7 ng/mL with or without losartan 10 μM, in the presence or absence of CBD 5 μM (**A**) or CBD 10 μM (**B**) for 24 h. NO levels were determined in the media and normalized to the cell number. Three independent experiments were performed, *n* = 12. ^aaa^ *p* < 0.001 vs. control (non-stimulated cells); ^b^ *p* < 0.05 vs. LPS; ^bbb^ *p* < 0.001 vs. LPS; ^ddd^ *p* < 0.001 vs. LPS + losartan; ^ccc^ *p* < 0.001 vs. LPS + CBD 5 μM; ^eee^ *p* < 0.001 vs. LPS + CBD 10 μM.

A synergistic inhibitory effect on NO production was also observed when cells were co-treated with losartan (10 μM) and CBD (5 μM) (decreased NO production by 80%) compared to cells exposed to each compound alone (by 22% and 26%, respectively) (Figure 2A). Co-treatment with increased concentrations of CBD (10 μM) produced an additive effect on LPS (7 ng/mL)-induced NO release (Figure 2B).

**Figure 3:**
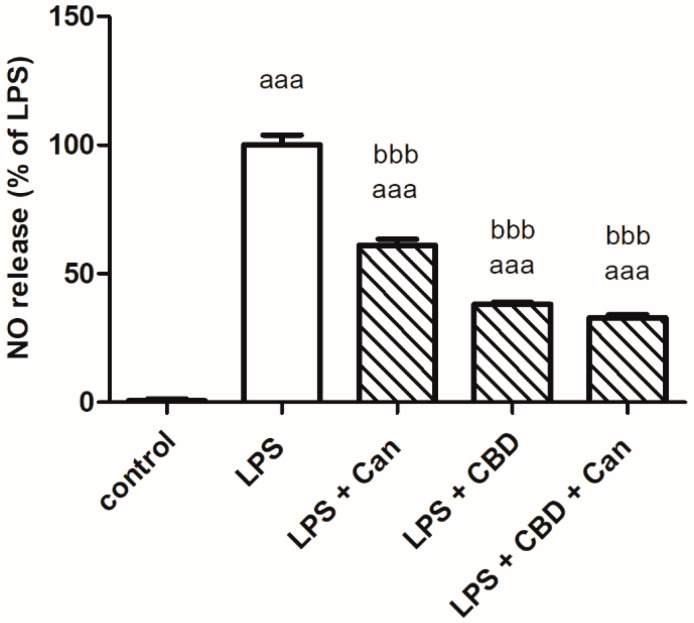
Candesartan and CBD given alone or together similarly decreased NO production in LPS-stimulated BV2 microglia. BV2 microglial cells were incubated with LPS 7 ng/mL, LPS 7 ng/mL + candesartan 5 μM, LPS 7 ng/mL + CBD 5 μM and LPS 7 ng/mL + CBD 5 μM + candesartan 5 μM for 24 h. NO levels were determined in the media and normalized to the cell number. Two independent experiments were performed, *n* = 8. ^aaa^ *p* < 0.001 vs. control (non-stimulated cells); ^bbb^ *p* < 0.001 vs. LPS.

As shown in Figure 3, the extent of the inhibition of NO production was similar when the combination of candesartan 5 μM and CBD 5 μM or only CBD was administered (decreased NO synthesis by 68% or 62%, respectively). A weaker inhibitory effect on NO synthesis was observed when candesartan was given by itself (decreased NO synthesis by 40%).

**Figure 4:**
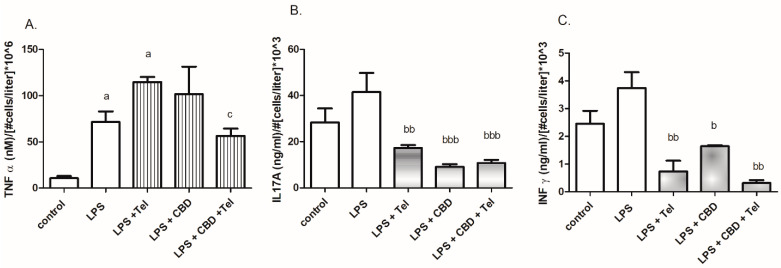
Cytokine determination of TNFα (**A**), IL17A (**B**) and IFNγ (**C**) following cotreatment with telmisartan and CBD in LPS-stimulated BV2 microglia. BV2 microglial cells were incubated with LPS 7 ng/mL, LPS 7 ng/mL + telmisartan 5 μM, LPS 7 ng/mL + CBD 5 μM and LPS 7 ng/mL + CBD 5 μM + telmisartan 5 μM for 24 h. Cytokine levels were determined in the media and normalized to the cell number. *n* = 12, three independent experiments were performed. ^a^ *p* < 0.05 vs. control (non-stimulated cells); ^c^ *p* < 0.05 vs. LPS + Tel; ^b^ *p* < 0.05 vs. LPS; ^bb^ *p* < 0.01 vs. LPS; ^bbb^ *p* < 0.001 vs. LPS.

In contrast, telmisartan but not CBD significantly increased (by 60%) LPS-induced TNFα release. Both telmisartan and CBD significantly reduced (by 22%) TNFα release (Figure 4A). A greater effect of CBD with or without telmisartan was shown relative to telmisartan regarding the attenuation of the LPS-induced interleukin 17A (IL17A) release (by 80%, 74%, and 60%, respectively) (Figure 4B). Greater inhibition of interferon γ (IFNγ) release in LPS-treated cells was observed in the presence of telmisartan with or without CBD (by 80% and 92%, respectively). CBD alone inhibited IFNγ release by 60% (Figure 4C).

**Figure 5:**
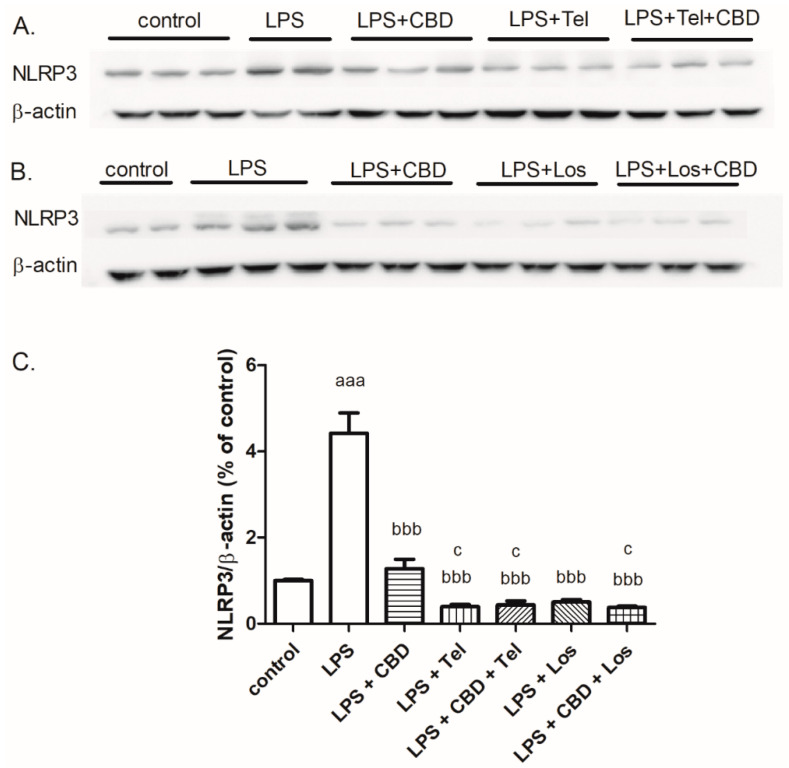
ARBs (telmisartan or losartan) and CBD, together or alone, decreased NLRP3 expression in LPS-induced BV2 microglia. BV2 microglial cells were incubated with LPS 7 ng/mL alone or together with CBD 5 μM or ARB (telmisartan 5 μM (**A**) or losartan 5 μM (**B**)) or both compounds for 24 h. Proteins from the cell lysate were loaded on 7.5% polyacrylamide-SDS gels. (**C**) An analysis of NLRP3 was performed using specific antibodies for it and β-actin. *n* = 6, two independent experiments were performed. ^aaa^ *p* < 0.001 vs. control (non-stimulated cells); ^bbb^ *p* <0.001 vs. LPS; ^c^ *p* < 0.05 vs. LPS + CBD 5 μM.

As shown in Figure 5, the 24-h treatment of BV2 cells with LPS resulted in a robust increase in NLRP3 inflammasome protein levels. A greater effect of telmisartan (5 μM) or losartan (5 μM) relative to CBD (5 μM), with or without CBD, by 89–92% was observed regarding NLRP3 inflammasome expression levels. CBD (5 μM) alone reduced NLRP3 expression by 73%.

**Figure 6:**
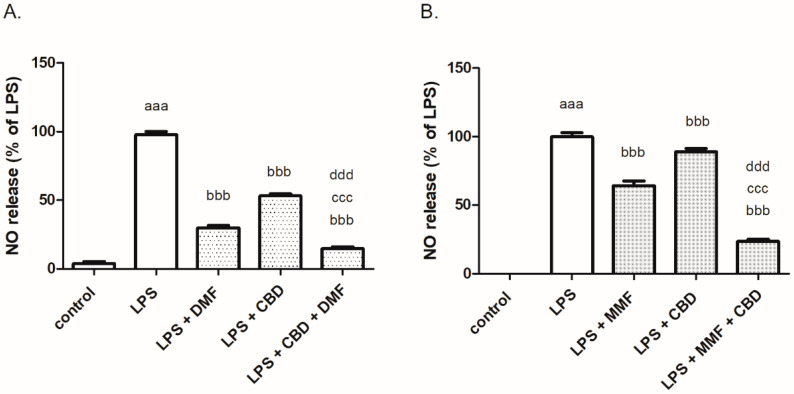
DMF (**A**) and MMF (**B**) together with CBD synergistically decreased NO production in LPS-stimulated BV2 microglia. BV2 microglial cells were incubated with LPS 7 ng/mL, LPS 7 ng/mL + DMF 5 μM or MMF 1 μM, LPS 7 ng/mL + CBD 5 μM and LPS 7 ng/mL + CBD 5 μM + DMF 5 μM or MMF 1 μM for 24 h. NO levels were determined in the media and normalized to the cell number. *n* = 8, two independent experiments were performed.^. aaa^ *p* < 0.001 vs. control (non-stimulated cells); ^bbb^ *p* < 0.001 vs. LPS, ^ccc^ *p* < 0.001 vs. LPS + DMF or MMF, ^ddd^ *p* < 0.001 vs. LPS + CBD.

DMF (5 μM) and CBD (5 μM) reduced NO release in BV2 cells by 71% and 47%, respectively, and co-treatment with both drugs resulted in the 86% inhibition of NO release (Figure 6A). MMF, the primary metabolite of DMF, and CBD synergistically decreased LPS-induced NO synthesis (by 78%) compared to the inhibitory effect of each compound alone (by 35% and 15%, respectively) (Figure 6B).

## 3. Discussion

This study highlights both the additive and synergistic (an interaction between drugs that causes the total effect to be similar to or greater than the sum of the effects of the drugs given alone) effects on glial NO levels. CBD (5 μM) was given with losartan (5 μM) or MMF (1 μM) to attenuate synergistically glial NO synthesis. Additive effects on NO production were observed when telmisartan (5 μM) and CBD (5 μM) were given together to glial cells.

This treatment could be used to modulate microglial inflammation, which plays a major role in regulating neurodegeneration.

In microglia, LPS is an inducer of the synthesis of inflammatory mediators and reactive oxygen species (ROS) [39]. It was shown that LPS-stimulated primary microglial cultures also displayed increased expression of the Ang II peptide and increased AT1R mRNA levels, which are targets of ARBs [40]. Ang II was characterized as an autocrine and paracrine mediator of brain inflammation, leading to neurodegeneration [41]. Taken together, these findings suggest that LPS and Ang II may lead to the increased release of various inflammatory factors, including cytokines and NO, which stimulate microglia and thus inflammatory cascades.

Previously, we investigated the effect of ARBs on the modulation of microglial inflammation. While LPS significantly increased NO production vs. non-treated cells (control), telmisartan attenuated this effect in a concentration-dependent manner [16]. Here, we confirmed and extended previous findings, showing that another ARB, losartan, slightly reduced LPS-induced NO synthesis in BV2 microglia. Our findings are in keeping with a previous in vitro study of primary rat microglia demonstrating that LPS-induced cytokine and NO production were significantly inhibited by losartan treatment [40]. A previous study documented similar inhibitory effects on cytokine release when LPS-stimulated microglia were exposed to a short-term (2 h) pre-treatment with CBD [15]. Here, we documented a 24-h inhibitory effect of CBD on microglial IFNγ, IL17A and NO production, suggesting changes in the levels of proteins responsible for the synthesis of these mediators. Interestingly, these drug-induced changes in inflammatory mediator concentrations were associated with a robust decrease in protein levels of the inflammatory pathway mediator, the NLRP3 inflammasome, when microglia were exposed to CBD or ARBs alone or in combination. Previous studies demonstrated that ARBs administered with or without CBD decreased LPS-induced NLRP3 expression by 80%. In BV2 cells, LPS also upregulated the expression of pro-inflammatory miRNAs related to Toll-like receptor (TLR) and NF-κB signaling. In contrast, CBD inhibits LPS-stimulated expression of these mRNAs [31].

As shown here, non-similar effects were observed for CBD when given with different ARBs. CBD acts as a partial agonist of PPARγR. Telmisartan, candesartan and losartan differ in their non-AT1R-mediated effects. For example, telmisartan and, to a lesser extent, candesartan activate PPARγR and inhibit neuroinflammation. Therefore, we hypothesize that the efficacy of combining CBD with ARBs will depend on the ARB type. We also hypothesize that the combined effect of CBD and ARBs will be additive or synergistic. This combination therapy has been attracting much attention recently, and new clinical studies are centered on this topic. Combination therapy aims to minimize the adverse effects and ideally produces a more efficient effect. This concept combines two therapeutic agents acting on different upstream pathways, but affecting similar downstream factors. Here, we show an in vitro additive or synergistic effect of telmisartan and CBD, as manifested by the attenuation of LPS-induced NO release from microglial cell lines (Figure 1 and Figure 5A). The combination of CBD and telmisartan showed no extra effects on IFNγ and IL17A release and NLRP3 expression. The effects on IFNγ release and NLRP3 expression are dominated by telmisartan and the effect on IL17A release is dominated by CBD. LBS-induced IFNγ and IL17A production, as well as NLRP3 expression, were significantly decreased by the combined treatment with telmisartan/losartan and CBD in BV2 microglia. Moreover, a synergistic inhibitory effect on LPS-induced microglial NO synthesis was also observed when the cells were treated with losartan and CBD compared to treatment with each compound alone.

Additive effects of CBD and ARBs may be explained by the ability of both treatments to modulate similar signaling pathways. For example, both inhibit the NF-κB pathway [15,42]. However, the synergistic effects of CBD and ARBs/MMF may be explained by the different pathways induced by these treatments. CBD is a G-protein-coupled receptor 55 (GPR55) antagonist, as shown by Nevalainen and Irving 2010 [43]. CBD also induces the suppression of autoimmune hepatitis, which was dependent on TRPV1 [44], and inhibits the uptake of the endocannabinoid anandamide (AEA) [45]. The latter effects are not shared by the ARBs. Further studies are required to elucidate the precise signaling pathways involved. An additional intriguing point was the potential therapeutic interest in the combined administration of DMF/MMF and CBD. DMF was approved as an effective treatment for multiple sclerosis (MS) in 2013 [46,47]. MMF, the primary metabolite of DMF, penetrates into the brain [48]. Both MMF and DMF combined with CBD significantly reduced NO synthesis compared to each compound alone. MMF but not DMF acted synergistically with CBD to inhibit NO production in LPS-stimulated microglia. MMF was demonstrated to exert relatively weaker effects than DMF, used at different concentrations, on NO release from macrophages as well [49] Although structurally similar, DMF and MMF may impact distinct cellular signaling pathways in microglia (e.g., DMF induced the activation of NRF2 and both DMF and MMF modulate the NF-κB pathway). This may lead to a distinct combined effect of each of compound with CBD.

In summary, this study demonstrates that CBD works synergistically in combination with ARBs or MMF to attenuate the synthesis of NO in LPS-induced microglia. Non-cannabinoid receptor-mediated mechanisms underly the efficacy of CBD in microglia [50]. Different mechanisms of action activated by ARBs or MMF may explain the synergistic effect shown upon the treatment of cells with both drugs. Further work is warranted to determine the mechanism of synergy between CBD and the compounds tested here at both the receptor and intracellular signaling levels, to enhance our ability to develop novel, safe and effective treatment strategies for microglial inflammation and neurodegeneration.

Further preclinical studies and clinical trials will be required to pave the way for possible clinical application.

## 4. Materials and Methods

### 4.1. Cell Cultures

The BV2 immortalized murine microglial cell line was constructed by infecting primary microglia with a *v-raf/v-myc* oncogene-carrying retrovirus (J2). BV2 microglial cells (murine) were kindly given to us by Professor Rosario Donato (Dep. of Experimental Medicine and Biochemical Sciences, University of Perugia, Italy). Cells were maintained in RPMI-1640 medium supplemented with fetal calf serum (10%), penicillin (100 U/mL), streptomycin (100 μg/mL) and L-glutamine (4 mM) in 5% CO_2_ humidified air at 37 °C. (All media components were purchased from Biological Industries, Kibbutz Beit-Haemek, Israel). The culture medium was replaced twice a week prior to experiments. Prior to each experiment, BV2 cells (passages 3–5 after thawing), were seeded in 24-well plates (3 × 10^5^ cells per well) or 6-well plates (1 × 10^6^ cells per well) and allowed to settle for 24 h. Serum-free medium (SFM) was added to the cells for 4 h prior to each experiment. Then, cells were treated with medium containing 0.1% bovine serum albumin (BSA), 2-[4-(2-hydroxy ethyl)piperazin-1-yl]ethanesulfonic acid (HEPES) buffer (10 mM), pH 7.4, and 1% serum (all were purchased from Sigma-Aldrich, Rehovot, Israel) with or without the test agents for the indicated periods of time. Telmisartan, losartan and candesartan were purchased from Tocris Biosience, Bristol, UK and lipopolysaccharide (LPS) and N,N-dimethylformamide (DMF) were obtained from Sigma-Aldrich, Rehovot, Israel. Concentrations and incubation times were taken from previous studies [16]. At the end of the experiments and normalization to the cell number, cells were harvested after incubation with 1 mL of SFM for 1 h at 4 °C and counted using a Z1 Coulter counter (Coulter electronics, Miami, FL, USA).

### 4.2. Determination of NO Levels (Griess Reaction)

BV2 cells were seeded onto 24-well plates (3 × 10^5^ cells per well). Tested agents were added for the indicated times. At the end of the experiment, medium was collected from each well for the NO determination. Nitrite levels (in culture supernatants), an indicator of NO release, were determined with an established assay using Griess reagent (Sigma-Aldrich, Rehovot, Israel). A standard curve of sodium nitrite was used. In total, 100 μL of culture supernatant and Griess reagent were mixed in a 96-well plate and incubated at room temperature for 15 min (in the dark). Then, the absorbance at 540 nm was measured using a microplate reader (model 680, Bio-Rad, Hercules, CA, USA). Cells were harvested in 4 °C SFM and counted using a Z1 Coulter counter (Coulter Electronics, Miami, FL, USA).

### 4.3. SDS-PAGE and Western Blot Analysis

Equal amounts of protein from the cell lysates were subjected to sodium dodecyl sulfate-poly acrylamide gel electrophoresis (SDS-PAGE). Proteins were separated on 7.5% polyacrylamide-SDS gels. Electrophoresis was performed in running buffer consisting of 25 mM Tris-HCl, pH 8.3, 192 mM glycine and 0.1% SDS in double-distilled water (DDW) and began by applying power to the Mini Protean II system at a constant voltage setting of 150 V for 75–90 min. Following SDS-PAGE separation, proteins were transferred to nitrocellulose membranes. Upon blocking (4% BSA, 90 min at room temperature), membranes were incubated for 24 h at 4 °C with a specific mouse anti-NLRP3 inflammasome antibody (1:1000; Adipogen, Milan, Italy). After washing, the blots were incubated for 90 min at room temperature with the corresponding-conjugated donkey anti-rabbit antibody (1:10,000; GE Healthcare, Little Chalfont, UK). The position of the individual protein was detected using an enhanced chemiluminescence (ECL) solution followed by exposure to X-ray film (Fuji medical X-ray film; FujiFilm, Tokyo, Japan). The band intensity analysis was performed using a computerized image analysis system (EZ Quant-Gel 2.2, EZQuant Biology Software Solutions Ltd., Tel Aviv, Israel). The protein quantity was normalized to β-actin protein level measurements conducted using a mouse anti-β-actin antibody (1:4000, Sigma-Aldrich, Rehovot, Israel) followed by exposure to a horseradish peroxidase-conjugated goat anti-mouse antibody (1:20,000; Immunoresearch Inc., West Grove, PA, USA).

### 4.4. Determination of Cytokine Levels

TNFα, IFNγ, and IL17A levels in the culture media were determined using enzyme-linked immunosorbent assay (ELISA) kits (BD Biosciences, San Diego, CA, USA) according to the manufacturer’s protocol.

### 4.5. Statistical Analysis

Experimental data are presented as the means ± SEM. For the assessment of significant differences between groups, one-way analysis of variance (ANOVA) and a post hoc multiple comparisons test (Tukey–Kramer multiple comparison test) were performed. Results are presented as means + SEM. Statistical significance was considered at *p* < 0.05.

## Data Availability

Data are contained within the article.
